# Investigating sport persistence through the development of the Sport Persistence Questionnaire

**DOI:** 10.1038/s41598-025-11231-3

**Published:** 2025-07-18

**Authors:** Karolina Eszter Kovács, Csilla Csukonyi

**Affiliations:** Institute of Psychology, Faculty of Arts, University of Debrecen, Debrecen, 4032 Hungary

**Keywords:** Sport persistence, Endurance, Sport, Ecological model, Human behaviour, Quality of life

## Abstract

Regarding sporting activity, one of the most important indicators of effectiveness may be sport persistence, a complex concept, a terminology referring to the mastery, form, level and efficacy of sporting activity. It encompasses our attempts concerning the performance plateau, failures, injuries, or even the resolution, processing and utilisation of stressful situations associated with success and positive events, combining the physical, mental and social aspects of sport. In our study, we aimed to describe the development of a tool to measure sport persistence. Accordingly, our research involved a multi-step process. Based on the experience of a preliminary research [N = 1165], a sport persistence principal component was created including four items, which was a reliable but not nuanced variable. To provide a nuanced picture of sport persistence, a 95-item long questionnaire was developed and tested during the pre-test [N = 1105], which was revised and analysed during the post-test [N = 212]). The results of both the pre-testing and post-testing phases indicated that the 13-item Sport Persistence Questionnaire (SPQ) instrument, which scores items on a five-point Likert scale, is reliable for measuring sport persistence (Cronbach’s α = 0.943). The indicators of the model fit were also found to be adequate. The instrument measures the level of sport persistence in one dimension. In conclusion, the findings of this study may be useful concerning tailored interventions in fostering sport persistence, applicable for coaches, educators and policymakers as well. The instrument provides a sound basis for further research. In the future, the investigation of sport persistence of competitive and recreational youth athletes will be available to detect the characteristics of sport persistence, including the qualitative, quantitative and longitudinal analysis of individual, micro, meso-, exo- and macro-level factors influencing sport persistence and intervention programs supporting persistent sporting behaviour.

## Introduction

In today’s sport, athletes or players do not pursue sport purely for pleasure, relaxation or recreation. Sports have become a complex and demanding profession that involves more strenuous training, early specialisation, careful planning, and the ability to prepare for and manage competitions and performance^1^. As players experience the pressures of managing training, work and other interests, this demanding situation puts more significant physical, psychological and social pressure on young athletes^2^. In many sports, such demands and requirements have led to the early dropout of many young, talented boys and girls^3^.

It is a fundamental fact that participation in sports positively impacts a person’s lifestyle, health and health awareness, regardless of gender and age^4,5^. Participation in sport from childhood promotes optimal psychophysical development and the acquisition of a healthy lifestyle maintained in later life^6,7^. Regular and optimally conducted sporting activities (i.e. not as an addiction) can develop psychological skills, including motivation, activity, self-discipline, perseverance, courage, willpower, self-confidence, pain tolerance or realistic self-assessment^8–10^. A key performance indicator may be sport persistence, a complex concept referring to sporting activity’s mastery, form, level, and effectiveness. Concepts and research focus primarily on sport motivation and commitment to sport, which are prominent components of sport persistence but do not cover the overall phenomenon of sport persistence going beyond it. It refers to our attempts concerning the performance plateau, to the resolution, processing and utilisation of stressful situations associated with failures, injuries or even successes and positive events, combining the physical, mental and social aspects of sport^11^.

Motivation is an umbrella term, i.e. a multifaceted and complex phenomenon, and its examination can have several aspects, in each case it depends on the way the question is posed which aspect is considered. It is based on need, linked to a state of deficiency. It may be physiological or higher, conscious or unconscious. The way in which the need is met is influenced by the environment, but also by prior experience^12^. Beyond regular physical activity, but based on it, engagement in sport refers to a higher level of engagement where a person, building on his or her strengths and overcoming his or her disadvantages, engages in active sporting activities^13^. As with regular sport participation, motivation is the cornerstone of sport engagement and determines the extent and quality of engagement^14,15^.

Overall, the notion of sport persistence, which entails an athlete’s resolute dedication to their chosen sport, is a combination of sports performance and commitment. It involves the cultivation of qualities such as resilience^16^, adaptive and proactive coping^13,16^ and positive personality characteristics that can also contribute to preventing dropout, and enabling athletes to show greater training commitment^17,18^. The athlete’s commitment goes beyond mere participation in the sport; it encompasses their ability to effectively navigate and utilize various challenges and circumstances, including performance setbacks, injuries, successes, and positive experiences^19^. Consequently, an athlete embodies a qualitative commitment to their sport, rather than simply being dedicated to the activity itself. The efforts we undertake to manage, understand, and navigate stressful circumstances linked to performance plateaus, setbacks, injuries, or even achievements and positive experiences also are incorporated into this term. In summary, based on the definition of sport persistence, it serves as a performance metric, reflecting an individual’s accomplishments through consistent physical activity, irrespective of the activity level^11,16,20^. Despite being an area that lacks extensive research in international practice, sport persistence plays a crucial role in an individual’s sustained engagement and performance in physical activity.

Gould and Horn indicate that annually, 33% of youths aged ten to seventeen withdraw from organized sports. This phenomenon impacts millions of young individuals across the globe^21^. High dropout rates in sports refer to significant challenges, including reduced long-term physical and mental health benefits for individuals. Various factors contribute to the decision of young people to leave sports, such as mismatches with teammates, insufficient success or anticipated progress in their sport, conflicts with coaches, a lack of enjoyment, among other considerations^22^. This trend also affects sports organizations and communities by limiting talent development and hindering the growth of participation programs. Furthermore, early disengagement from sports can perpetuate negative lifestyle habits and discourage future involvement in physical activity, contributing to broader public health concerns. To effectively address dropout rates, it is essential for sports professionals involved in youth athletics to thoroughly understand these causes, enabling them to create sports programs and experiences that cater to the athletes’ needs and foster their personal growth positively^23^. Addressing these rates is crucial for promoting lifelong engagement in sports and maximizing the benefits of physical activity.

Young athletes, especially the talented ones, may, due to personal, social and contextual factors, drop out of sport prematurely during their schooling before they reach their potential peak performance^3,24^. The combination of gender, socio-economic, and peer (parents, coaches, peers) supporting factors may predict sport persistence and its opposite, dropout from sport, in childhood^25,26^. Therefore, both sport persistence and dropout have been identified as multifactorial and complex phenomena, strongly influenced by different sociocultural backgrounds and behavioural factors, as well as personal characteristics, types of sport, attitudes and motivations^27^. Bronfenbrenner’s socio-ecological model^28^, as a general theory may be applied to explain differences in individual sport participation and persistence. The basic premise of Bronfenbrenner’s socio-ecological model is that individuals are closely connected to and influenced by their environment. In particular, Bronfenbrenner argues that individual behaviour can be understood by analysing the four environmental systems known as the micro-, meso-, exo- and macro-system. These different systems are layers of nested systems (like a matryoshka doll set), with the innermost layer representing the self. First, the micro level consists of a complex of close relationships, such as with family members, at work, in the classroom, in the neighbourhood, and with peers. The meso-system represents the second layer. This is the context in which micro-systems such as family, neighbourhood, and school are interconnected. The meso-system, therefore, refers to the relationships between micro-systems.

The adapted ecological model of Bauman^29^ expands the list mentioned above. Regarding the individual level, the model counts with psychological (cognition, beliefs and motivation) and biological (genetic and evolutional physiological) factors that are key to maintaining sport engagement. Individual psychological variables underlying sport participation are highly complex and multifaceted. The importance of positive psychological variables (i.e. adaptive coping, positive self-esteem, motivation and goal orientation, resilience, engagement, positive emotions) as preventive factors against drop-out from sport should be emphasized^2,27,27,30,31^. At the interpersonal stage, social support (including family, friends and work) and cultural norms and practices appear. The family serves as the foremost environment for socialization, offering both emotional and practical support that is crucial during childhood and in competitive sports^4,32^. Additionally, relationships with peers can foster a sense of camaraderie, encouragement, and a collective purpose, all of which enhance an athlete’s motivation and dedication. Through shared experiences, collaboration, and feedback, peers create opportunities for personal growth and learning^4,33,34^. Furthermore, coaches are indispensable in this process; their role is critical in skill enhancement and delivering constructive feedback. When coaches offer effective guidance, technical knowledge, and insightful input, athletes can elevate their performance, thereby boosting their motivation and determination^35,36^. Regarding the environment, Bauman reflects on the social environment (behavioural modelling; crime, traffic, incivilities; organisational practices), the built environment (community design; neighbourhood walkability; public transport; parks and recreation facilities; aesthetics and pleasantness; walking and cycling facilities; building location and design; pedestrian safety; crossings) and the natural environment (vegetation, topography, weather; national parks, trails, walking routes). Adequate sports infrastructure is important for sport, as many sports activities cannot be practized without adequate sports facilities. Sporting facilities can be a guarantee of development, as they can facilitate personal development, becoming a top athlete, and physical and mental preparation^37–39^. Concerning the regional and national policy, transport systems, urban planning and architecture, parks and recreation sector, health sector, education and school sector, organized sports sector, national physical activity plans, national physical activity advocacy, and corporate sector are mentioned. Efficient and accessible transport systems make it easier for individuals to reach sports facilities, recreational areas, and organized sporting event. Thoughtful urban planning that prioritizes the creation of spaces conducive to physical activity can also encourage regular engagement in sports^37,40^. Lastly, economic development, global media, global product marketing, urbanisation, global advocacy, and social and cultural norms are determined at the global level. Economic development may allow more investment in infrastructure, including sports facilities, transportation, and community programs^41^. The global media plays a major role in promoting sports by increasing visibility and creating role models^42^. Socioeconomic factors (e.g. cost, quality and accessibility) can also influence physical activity choices, as membership (e.g. health/fitness clubs), registration fees, equipment and transport can limit activity opportunities for economically disadvantaged people^43,44^.

The study aims to explore the methodological possibilities of sports persistence through the example of test development. The shortcoming of the existing research is that sport persistence is considered a bivariate variable. Thus, athletes are included in the persistent group if they are still playing sports at the time of the study, i.e. have not dropped out. However, capturing persistence more precisely by creating a complex indicator would be essential. Therefore, in our studies, our aim was to create a questionnaire specifically focusing on the sport persistence of athlete. We designed a three-step research process focusing on the development, test and re-test of the psychological construct. During the research, we also set the following research questions:

1. *What kind of measure can we use for exploring sports persistence?*
2. *How can we measure the phenomenon of sport persistence in a reliable way?*

Answering these questions and creating a single questionnaire allow us to measure the nature of sport persistence and factors influencing its manifestation (e.g. along with Bauman’s model) in detail.

In our research, we aimed to create a psychological measure that can be used to assess sport persistence. Our test development process included three steps. First, we created a principal component from bivariate variables before developing the test in our pilot study. As a second step, we created a lengthy questionnaire, which was tested and shortened to a more reliable measure. Lastly, a post-test and reliability analysis were carried out.

In our analyses, we used IBM SPSS 22.0 and Jamovi 2.2.5.0 statistical softwares. As statistical analysis, principal component and exploratory and confirmatory factor analysis (Maximum likelihood, varimax rotation) and reliability indicators, i.e. Kaiser–Meyer–Olkin (KMO), Bartlett-test, Cronbach alpha, Chi-square (χ2), degree of freedom (df), Comparative Fit Index (CFI), Tucker–Lewis Index (TLI), Root Mean Square Error of Approximation (RMSEA), Standardised Root Mean Square Residual (SRMR) were used.

## Study 1—preliminary research

### Sample

Our preliminary research used data from a large, representative database, PERSIST 2019 (conducted by the Center for Higher Educational Research and Development, CHERD, led by Professor Gabriella Pusztai), which is based on a quantitative survey. The study sample includes students from higher education institutions in five countries (Hungary, Slovakia, Romania, Ukraine and Serbia). The cross-border sample is composed using probability sampling (N = 1165). The majority of participants are full-time second-year BA/BSc students and second or third-year MSc students, although in some cases, due to low student numbers, students from upper years were also included in the response. Students involved in the study should have reported pursuing sport at least once per week. Overall, inclusion criteria of the study included being university students learning in the Central Eastern European Region engaging in regular physical activity (at least once a week). Those who did not meet this criterion were excluded.

29.9% of respondents were male and 70.1% female. The mean age of the sample is 22.97 years (SD = 2.84). 2.6% of respondents lived in the capital, 25.2% in a county or city with county status, 34.4% in a smaller town and 37.7% in a village, based on their permanent place of residence at age 14 (the survey asked about the type of settlement at age 14). 34.7% of the respondents’ fathers/carers had primary education, while the proportion of fathers with secondary education was 37.9% and 27.4% with tertiary education. Regarding maternal education, 26.3% of respondents had primary education, 39.9% had secondary education, and 33.7% had tertiary education.

### Methods

The PERSIST 2019 database contains data on various research areas, such as academic achievement, motivation to learn, health awareness, sporting habits, and volunteering. As the present research aims to investigate sport persistence, a sport persistence principal component was created based on the database questions that refers to past and ongoing successful sporting activities. In this case, principal component analysis was used to create a variable that refers to the phenomenon. It integrates the following elements:

- whether a student has won a sports scholarship during their higher education studies
- whether the student received extra credit for his/her sporting performance during the admission procedure
- whether the student is a member of a sports club or association
- the student is currently playing sports.

### Results

The variable created by principal component analysis, therefore, comprises four variables. Since the variables have the same response options, they should be interpreted along the same scale. Therefore, no standardisation was necessary. The communality of the four variables is sufficient; all four variables can be retained as components of the principal component (Table 1).

**Table 1:** Communalities of principal component variables.

	Communalities
Having any sports scholarships	0.460
Having extra points for sporting performance	0.476
Being a member of a sports club/association	0.413
Pursuing any kind of sport	0.359

The total variance explained is not very high, 42.662%, which still just allows us to keep our principal component. The KMO is 0.670, which is an acceptable value. The Bartlett test also allows us to retain the principal component (χ^2^ = 531.797; df = 6; p < 0.001). Extra points received for sports performance had the highest weight, followed by the award of a sports scholarship, then by the membership of a sports club or association, and finally by the fact of playing sport. The factor weights are illustrated in Table 2.

**Table 2:** Factor weights of principal component variables.

	Factor weights
Having extra points for sporting performance	0.690
Having any sports scholarships	0.678
Being a member of a sports club/association	0.642
Pursuing any kind of sport	0.599

The resulting principal component values were recoded to a 100-degree scale using the IBM SPSS Syntax Editor to ease the interpretation. The minimum value is 0 points, and the maximum is 100 points. The sample mean is 87.48 points, and the standard deviation is 16.13.

## Study 2—The first phase of test development

The preliminary study shows that the principal component based on the four variables meets the necessary criteria based on the parameters measured in the principal component analysis. Thus, an objective indicator of sport persistence has become available and applicable. However, it is necessary to point out that the principal component generated is based on bivalent variables, which cannot provide a nuanced picture of sport persistence. On the other hand, the variables allow us to infer an objective, factual indicator that does not reflect the athlete’s experience. Thirdly, the literature review shows the complexity of sport persistence, so it is worth reflecting on whether the four questions can reflect an individual’s level of sport persistence. Therefore, using the previous research results and theoretical knowledge on the topic, we created a longer and more extensive questionnaire to detect the individual’s level of sport persistence.

### Sample

The created questionnaire was tested on a sample of 1105 people using a snowball design. To ensure the goal of the research, during the sample size calculation, we considered the recommendations of MacCallum et al.^45^ who reports that at least 5–10 participants per item are recommended, with a minimum sample size of 100 participants to ensure robust factor analysis results. Sports clubs were asked to support the distribution of the questionnaire and online (social media) platforms were also used for this reason. Following our inclusion criteria, university students pursuing regular physical activity were involved in the research. As inclusion criteria, participants had to be university students learning in a Hungarian higher educational institute, engaging in regular physical activity (at least once a week). Those who did not meet this criterion were excluded. Only healthy participants were considered, and having any kind of illnesses was considered exclusion criteria. Both competitive and recreational athletes had the opportunity to participate in the survey to ensure diversity in terms of sport type, gender, or socio-economic background. Taking part in sporting activities less than once per week was also regarded as exclusion criterium.

22.5% of the respondents were male and 77.5% were female. The mean age of the sample is 22.03 years (SD = 2.11). By type of settlement, 15% of the respondents live in the capital, 20% in the county capital, 22.5% in a large city, 7.5% in a small town and 35% in a village. Regarding parents’ educational attainment, 67.5% of respondents’ mothers have a secondary education, while 32.5% have a tertiary education. Regarding the father’s education, 2.5% of respondents have a primary education, 72.5% have a secondary education, and 25% have a tertiary education. 90% of the respondents are recreational athletes while 10% are professional athletes (respondents who reported not pursuing sports regularly were excluded from the study). Most respondents (72.5%) play an individual sport, 20% play a team sport, and 7.5% do not pursue a sport regularly.

### Methods

To create a broad, multidimensional tool that covered both objective indicators (e.g., participation frequency) and subjective experiences (e.g., resilience, motivation), an initial 95-item questionnaire was developed from a comprehensive literature review on sport persistence, sport motivation, and dropout from sport. This extensive starting point ensured that all potentially relevant aspects of sport persistence were captured, aligning the questionnaire with established theoretical frameworks (e.g., Bronfenbrenner’s and Bauman’s socio-ecological model). The statements of the questionnaire are rated on a 5-point Likert scale, where 1 means "not typical at all" and 5 means “very typical”. The following instruction was given to the participants: “Below you will find statements about your sporting activities. Please read them carefully and select how typical the following statements are for you (1 = not at all, 5 = very typical)”. IBM SPSS 22.0 statistical software was used for the analyses.

### Results

In testing the questionnaire, we first looked at the reliability, which is very high, with a Cronbach’s α of 0.969. However, exploring the questionnaire’s factor structure was also deemed necessary, a mandatory step in designing questionnaires. For this purpose, principal component analysis was used. This analysis showed that the items’ communality was adequate (since the values were higher than 0.250 in all cases). However, the principal component analysis revealed 20 principal components, which fragmented the questionnaire too much. The total variance explained by the 20 principal components is 90.327%.

Principal component analysis allowed us to reduce the initial 95-item questionnaire into a smaller, more manageable set of items while retaining the most meaningful variance in the data. This is a critical step in scale development to identify the underlying structure among numerous variables. The objective was to simplify the measurement of sport persistence without losing conceptual depth. By identifying principal components, we aimed to ensure that the retained items effectively captured the core aspects of sport persistence. Thus, although the questionnaire appeared reliable, the principal component analysis did not reveal any item relationships that would have allowed precise and accurate sub-dimensions to be constructed for the questionnaire. Therefore, regarding reliability indicators, we looked at how the reliability of the questionnaire would evolve if certain items were removed from our questionnaire. However, the calculations did not lead to any significant improvement in this respect, as the deletion of certain items would not have improved the reliability of the questionnaire, given the high reliability already introduced. Therefore, as a selection procedure, inter-item correlations were used to see which questions had too high a coincidence to allow for the deletion of certain items.

Based on the results of the inter-item correlations, we performed a significant item reduction, retaining 13 of the original 95 items. The 13 remaining items were again subjected to principal component analysis, leading to more transparent and valuable results. The 13 items are grouped into one principal component. The communality of the items is appropriate in all cases, as illustrated in Table 3.

**Table 3:** Communalities of a reduced number of items.

Items	Extraction
I am very persistent in my sporting activities	0.546
I use several specific strategies to maintain regular exercise	0.550
With enough effort and practice, I can do well in most sports	0.488
I am very determined to continue this sport	0.759
I cannot imagine my life without sports	0.673
Future competitions and events will strengthen my commitment to the sport	0.553
I would continue pursuing sport after an injury (after recovery)	0.608
I will not let external factors hinder my sporting activities	0.628
I am constantly trying to improve myself in this sport	0.675
I spend a lot of time training in this sport	0.710
I put a lot of physical effort into my sport	0.588
I put a lot of mental effort into my sporting activities	0.544
I feel that all the energy I put into sports is paying off	0.480

The KMO value is 0.855, which is close to excellent, and the Bartlett test results are also good (χ^2^ = 367.481; df = 78; p < 0.001). The explained variance is 60.017%, which is lower than that of the 95-item questionnaire but still has a reasonable level of explanatory power. The factor weights of the items are illustrated in Table 4. All items have a high weight in the principal component, with almost all having weights close to or above 0.7.

**Table 4:** Factor weights for a reduced number of items.

Items	Factor weight
I am very determined to continue this sport	0.871
I spend a lot of time training in this sport	0.843
I am constantly trying to improve myself in this sport	0.821
I cannot imagine my life without sports	0.820
I will not let external factors hinder my sporting activities	0.792
I would continue pursuing sport after an injury (after recovery)	0.780
I put a lot of physical effort into my sport	0.767
Future competitions and events will strengthen my commitment to the sport	0.744
I use several specific strategies to maintain regular exercise	0.741
I am very persistent in my sporting activities	0.739
I put a lot of mental effort into my sporting activities	0.738
With enough effort and practice, I can do well in most sports	0.699
I feel that all the energy I put into sports is paying off	0.693

Based on the reliability test results, the Cronbach’s α of the new 13-item questionnaire was 0.943. The minimum value on the overall index was 13 points, and the maximum value was 65 points. The sample mean was 12.36 points (SD = 12.36). The questionnaire was considered reliable and applicable in this form, so the next step was to re-test it on a larger sample.

## Study 3—Re-test phase

### Sample

The new sample collected for re-testing the measure was created with snowball sampling (N = 212). To ensure the goal of the research, we followed the same recommendations of MacCallum et al.^45^. Similar to Stage 2, our inclusion criteria allowed to involve university students (studying in a Hungarian higher educational institute) pursuing regular physical activity (at least once per week). Taking part in sporting activities less than once per week was regarded as exclusion criterium. Only healthy participants were considered, and having any kind of illnesses was considered exclusion criteria. Both competitive and recreational athletes had the opportunity to participate in the survey. Sports clubs were asked to support the distribution of the questionnaire and online (social media) platforms were also used for this reason to ensure diversity in terms of sport type, gender, or socio-economic background.

38.4% of the respondents are male and 61.6% are female. The mean age of the sample is 21.95 years (SD = 3.78). 6.3% of the respondents live in the capital city, 42.9% in the county seat, 17% in a larger city, 13.4% in a smaller town, 16.1% in a village and 4.5% in a farm. 49.9% of the respondents have a father with secondary education, while 50.1% have a higher education. Regarding the mother’s education, 1.8% of respondents have primary education, 42% have secondary education, and 56.3% have tertiary education.

### Methods

IBM SPSS 22.0 and Jamovi 2.2.5.0 statistical software were used for the analyses. After refining the questionnaire to 13 items, confirmatory factor analysis was applied to check the structure of the questionnaire, expecting that the data would be clustered into a single factor, similar to the pre-test. Confirmatory factor analysis confirms whether the data fits the expected factor model, which is crucial for validating the construct. To set cutoff scores, the Border group methodology of Livingston and Zieky^46^ was applied. In this case, judges categorize individuals based on their response rates, identifying them as having either low or high levels according to the assessed trait. The individuals at the minimum level of high persistence which includes individuals who are in the middle of the two classifications, that is, those who own neither low nor high response level, are considered as the border group. The cutoff for this method was determined by determining the median of the rater’s scale scores. To establish the cut-off score for this approach, the median of the judges’ scale scores is calculated.

### Results

We obtained results as expected, with a KMO of 0.925, which is considered an excellent value. Bartlett’s test results are also satisfactory (χ^2^ = 1064.788; df = 78; p < 0.001). The communality of the items is adequate; thus, we can keep them as components of the questionnaire. Therefore, confirmatory factor analysis provided robust statistical validation that the reduced set of items formed a coherent, one-dimensional measure of sport persistence. This confirmation ensured that the final version of the Sport Persistence Questionnaire (SPQ) accurately reflects the construct it intends to measure (Table 5).

**Table 5:** Communality of the items of the finalised questionnaire.

Items	Initial	Extraction
I am very persistent in my sporting activities	0.684	0.649
I use several specific strategies to maintain regular exercise	0.598	0.463
With enough effort and practice, I can do well in most sports	0.486	0.379
I am very determined to continue this sport	0.687	0.643
I cannot imagine my life without sports	0.694	0.667
Future competitions and events will strengthen my commitment to the sport	0.381	0.306
I would continue playing sport after an injury (after recovery)	0.635	0.518
I will not let external factors hinder my sporting activities	0.611	0.550
I am constantly trying to improve myself in this sport	0.751	0.753
I spend a lot of time training in this sport	0.743	0.718
I put a lot of physical effort into my sport	0.781	0.709
I put a lot of mental effort into my sporting activities	0.680	0.578
I feel that all the energy I put into sports is paying off	0.573	0.555

The explained variance is 60.745%, almost the same as the pre-test explained variance. The factor weights of the items are illustrated in the table below. It can be seen that all items have a high weight in the principal component, almost all of them with weights close to 0.7, some with weights higher than 0.7 (Table 6).

**Table 6:** Factor weights for the items of the finalised questionnaire.

Items	Factor weights
I am constantly trying to improve myself in this sport	0.865
I put a lot of physical effort into my sport	0.853
I spend a lot of time training in this sport	0.849
I cannot imagine my life without sports	0.829
I am very persistent in my sporting activities	0.815
I am very determined to continue this sport	0.814
I put a lot of mental effort into my sporting activities	0.788
I will not let external factors hinder my sporting activities	0.777
I feel that all the energy I put into sports is paying off	0.772
I would continue playing sport after an injury (after recovery)	0.747
I use several specific strategies to maintain regular exercise	0.725
With enough effort and practice, I can do well in most sports	0.659
Future competitions and events will strengthen my commitment to the sport	0.587

In addition to adequate explanatory power, reliability testing also led to mixed results. The test resulted in a Cronbach’s α of 0.942, which is almost identical to the value obtained in the pre-test. The indicators of the model fit were found to be adequate (χ^2^ = 15.157; df = 65; CFI = 1.000; TLI = 1.037; RMSEA = 0.000; SRMR = 0.049). Since the questionnaire structure is stable and the questionnaire itself is reliable, it is considered suitable for use in future research.

To set the cut-off score, we measured the median of sport persistence, which was 52 points on the sample. However, it is necessary to distinguish between competitive and recreational athletes. Therefore, we set two cu-off scores for sport persistence based on the level of sporting activity. For competitive athletes, the median was 55 (M = 53.6, SD = 8.7) while for recreational athletes, the median was 47 (M = 45.5, SD = 12.5). Therefore, the cut-off score for competitive athletes was 55 points, meaning that competitive athletes scoring less than 55 points are in a potential risk of dropping out of competitive sport. Meanwhile, the cut-off score for recreational athletes was 47 points, meaning that non-competitive athletes scoring less than 55 points are in a potential risk of dropping out of sport.

## Discussion

Sport persistence is a less widely used terminology in the literature, which can be considered the peak of sport performance and commitment, referring to the active and sustained engagement and persistence in sporting activities, as reflected in objective and subjective performance and behaviour^4,11^. As research has focused primarily on sport motivation and commitment to sport, sport persistence is less manifested in theoretical concepts and research, and little is known about its characteristics. We can mainly conclude from the results of engagement and dropout studies, which can only be indirect evidence^27,29^. In our study, we have presented a process of multi-stage test development based on the main characteristics of sport persistence, including intergroup heterogeneity and correlations along some basic sociodemographic variables.

In our preliminary research, we created a sport persistence indicator based on questions from a large sample representative database of higher education students, which was based on four questions indicating active and persistent sporting behaviour (sports scholarship, extra points for sports performance during higher educational entrance examination, sports club/sports association membership, current sports participation) using principal component analysis. The principal component generated had sufficient explanatory power and proved suitable for further calculations. Based on the questions, this variable serves the role of an objective indicator of sport persistence, as the incorporated questions are themselves objective indicators of performance. In this capacity, the question set is therefore well suited to measuring objective sport persistence.

The above objective indicator is an improvement over existing research, where, in most cases, sport persistence is assessed by answering an objective question (whether currently continuing or dropping out). However, it would be important to develop a measure of subjective performance and behaviour alongside the objective performance components, which was the primary aim of our research. Preliminarily, seeing the amount of complex potential impact factors, it was expected that this could be done by creating a longer, more complex questionnaire. However, in the first stage of test development, it became clear that a similar questionnaire would fragment the factors too much, making interpretation difficult, and these were not always correlated with the expected factors. It was, therefore, decided to reduce the number of items significantly (from 95 to 13) by using reliability indexes and inter-item correlations. This reduced the instrument’s items to a principal component, which does not dimensionalise the effect factors but holds its own as an individual-oriented subjective indicator. The results of the testing on 212 participants are in line with the results of the pre-testing. The confirmatory factor analysis results show that the items were clustered into a single factor, as in the pre-testing, and the instrument was found to be adequate both in terms of reliability indicators and fit indicators. Confirmatory factor analysis provided robust statistical validation that the reduced set of items formed a coherent, one-dimensional measure of sport persistence. This, along with the high reliability of the instrument, ensured that the final version of the Sport Persistence Questionnaire (SPQ) accurately reflects the construct it intends to measure. Strong fit indices also demonstrated that the final model accurately represents the data structure, supporting the validity of the questionnaire. Finally, each retained item reflects a distinct, critical aspect of sport persistence. The reduced set demonstrated strong statistical properties. Cronbach’s α of 0.943 indicates excellent internal consistency and high reliability. All items had high factor loadings (most close to or above 0.7), indicating they strongly contribute to the principal component of sport persistence. Lastly, good fit indices: also indicate excellent model fit.

The primary objective of this research was to test a developed psychological construct. The goal set at the beginning of the research, to create a measurement tool that reliably and independently (not as part of another construct) measures sport persistence, has been achieved. The final 13-item version of the SPQ provides a balanced, reliable, and practical tool. It captures the essential elements of sport persistence without overwhelming respondents or researchers with redundancy. This refinement process ensures that the SPQ is both theoretically grounded and methodologically sound, enhancing its usability in future studies. This study aimed to address a gap in the current research, which only considers sport persistence as a bivariate variable^10,16^. We believe that sport persistence should be regarded as a spectrum instead of categorising athletes as persistent if they are still actively participating in sports at the time of the study, without dropping out.

The strength of the research lies in the multi-stage test development process, which was carefully and thoroughly planned and implemented. A weakness of the research is the specificity of the sample, as this research used a snowball sample and did not aim to be representative. The research had to meet two essential criteria: age (university student population) and regular sporting activity.

The findings of this study may be useful concerning tailored interventions in fostering sport persistence, highlighting its role as a complex, multidimensional construct encompassing physical, psychological, and social factors. Beyond academic contexts, these results hold significant applications for practitioners such as coaches, educators, and policymakers. Coaches and sport organisations can use the Sport Persistence Questionnaire to identify athletes who may be at risk of dropout and implement personalised strategies to build resilience and maintain motivation. The SPQ’s insights into persistence factors can guide the development of training programs that address individual needs, incorporating techniques to navigate challenges like injuries or performance plateaus^47,48^. The measure may be valuable for educators too, who can use the tool in school-based physical education programs by emphasizing persistence-related skills, such as goal setting, resilience, and self-regulation. These programs can foster not only athletic growth but also broader life skills, encouraging students to apply persistence across academic and personal domains^49,50^. Policymakers have the opportunity to use the SPQ to guide the allocation of resources and the development of community sports programs. By tackling systemic obstacles like limited access to facilities or socioeconomic challenges, they can foster equitable involvement and sustained engagement in sports^51^.

While this study provides valuable insights into sport persistence and successfully develops a reliable measurement tool, some limitations should be mentioned to provide a balanced perspective. Non-representative samples were used during the research, relying on a snowball sampling method which limits the generalisability of the findings, as it may not accurately reflect the broader population of athletes across diverse geographic, socioeconomic, and cultural backgrounds. Future studies should aim to employ random or stratified sampling methods to enhance representativeness. The current study focused on university students engaging in regular sporting activities which excluded other significant groups, such as younger athletes. As a result, the findings may not capture the full spectrum of sport persistence, particularly in individuals facing barriers to sustained engagement. Besides, the cross-sectional design restricts the ability to draw causal inferences about the factors influencing sport persistence. Longitudinal research is needed to explore how persistence evolves over time and the interplay of environmental, psychological, and social factors in shaping it. Addressing these limitations in future research could strengthen the validity of the findings, expand their applicability, and provide deeper insights into fostering long-term persistent sporting activity.

Our research can provide a reasonable basis for further research on sport persistence. Our further aim is to investigate sport persistence in competitive and recreational high school and university students, to detect the characteristics of sport persistence, to explore the factors involved in its development fully, and to apply the research results in practice. As a first step, the factors involved in developing sport persistence will be detected through qualitative research methods (interviews)^52,53^. Subsequently, we plan to explore the different contributing to sport persistence in a nationally representative study based on Bronfenbrenner’s^28^ and Bauman’s^29^ ecological models, thus focusing on sociodemographic and sporting habits, as well as on the role of individual, micro, meso-, exo- and macro-level factors. Further studies may also focus on conducting longitudinal research to measure the manifestation of sport persistence over time. This would help identify how persistence evolves and what kind of intra- and interpersonal and environmental factors contribute to persistent sporting behaviour. Qualitative and quantitative research results may be applied to design targeted intervention programs. These programs should aim to foster sport persistence by addressing common barriers (e.g., injuries, lack of motivation) and promoting facilitators (e.g., resilience, social support). Our long-term goal is to build on the experience of qualitative and quantitative research to create a sports persistence training program that supports adolescents’ and young people’s sport persistence, focusing on intrinsic strengths based on the correlates identified in this research. Although it is proven that the efficacy of such programs is an underinvestigated research topic, these training programs can provide individuals with the opportunity to develop and improve their athletic skills. Concerning the psychological skill-related aspect, persistence programs can encourage consistent effort, athletes can learn how to set both short-term and long-term objectives. They can help shape individuals’ character by fostering important qualities such as discipline, perseverance, teamwork, and resilience, leading to long-term engagement. Last, but not at least, sports persistence programs often foster a sense of camaraderie and community among participants^54^. We believe that our instrument may have an added value in the development and assessment of sport training programs.

## Data Availability

Data are available only on request due to ethical restrictions. For further information, please contact the corresponding author, Karolina Eszter Kovács PhD (kovacs.karolina@arts.unideb.hu).
